# Turkish Validation of the User Version of the Mobile Application Rating Scale

**DOI:** 10.5152/tud.2022.21324

**Published:** 2022-05-01

**Authors:** Gokhan Calik, Betul Bersan Kartal, Stoyan Stoyanov, Stavros Gravas, Lavin Othman, Jean de la Rosette, Selami Albayrak, Pilar Laguna

**Affiliations:** 1 Department of Urology, Medipol Mega University Hospital, İstanbul Medipol University. Istanbul, Turkey; 2 Queensland University of Technology Faculty of Creative Industries, Education & Social Justice, School of Design, Brisbane, Australia; 3 Department of Urology, University of Thessaly Faculty of Medicine, School of Health Sciences, Larissa, Greece; 4 Department of Urology, Professor of Medical School, University of Cyprus, Nicosia, Cyprus; 5 Department of Urology, İstanbul Medipol University Faculty of Medicine, İstanbul, Turkey

**Keywords:** mHealth, mobile health, mobile application

## Abstract

**Objective:**

**:** As the number of mobile health applications increases, quality assessment becomes a capital feature of any mobile application design. Besides the professional evaluation conducted before marketing the app, the perceptions of the subjects to whom is intended will determine the successful widespread dissemination. Hence, the implementation of a given app may be impaired by the lack of a validated translation and cross-cultural adaptation. We aimed to validate in the Turkish language the User Version of the Mobile Application Rating Scale, an English original scale designed to assess the quality of mobile health applications.

**Materials and methods:**

A well-established and predefined process of cross-cultural adaptation and translation to Turkish of the User Version of the Mobile Application Rating Scale according to the World Health Organization guidelines was performed using a common, readily available, free-of-charge application. Internal consistency and reliability were tested in a population sample by Cronbach’s *α* and *r*_WG_ index, respectively.

**Results:**

The total User Version of the Mobile Application Rating Scale score had good internal consistency (Cronbach’s *α* = 0.87). Internal consistencies of its subscales were also acceptable: with Cronbach’s *α* of 0.71, 0.78, 0.71, and 0.73 for engagement, functionality, aesthetics, and information, respectively. Cronbach’s *α* of the satisfaction subscale was 0.46. The User Version of the Mobile Application Rating Scale total and subscales scores had a strong within-group agreement, all of them with *r*_wg_ indexes between 0.78 and 0.87 over baseline to 1 month.

**Conclusion:**

The Turkish version of the User Version of the Mobile Application Rating Scale is consistent with the English original version and is a reliable and valid tool to assess the quality of mobile applications by Turkish users.

**Main Points**

- The User Version of the Mobile Application Rating Scale (uMARS) version of the Turkish language provides a reliable instrument to evaluate the general quality of applications (apps) in the Turkish language.
- uMARS is an easy-to-use questionnaire by untrained mobile app end-users.
- As the medical mobile app market rapidly grows in Turkey, the valıdated Turkish versıon of the uMARS represents a useful tool to assess future app qualıty.

## Introduction

Presently, there are more than 7 billion mobile subscribers in the world.^1^ In 2017, there were more than 350 000 mobile health (mHealth) applications (apps) available in the app stores and at least 19% of mobile users have downloaded health-related apps on their smartphones.^2^ While healthcare professionals are increasingly using mHealth apps as a source of information, diagnostic help, and in dynamic clinical scenarios to facilitate monitoring patient medication and symptom management, the primary target population for mHealth apps is the users. As of 2015, 82% of the US individuals aged 18-49 years owned an app-enabled mobile phone, and cross-sectional studies show that almost a third of the mobile users had downloaded a health-related app, mostly related to fitness and nutrition.^3-5^ These figures underscore the magnitude and impact of mHealth apps in the current e-health environment.^6^

The rapidly evolving mHealth app market leads to an overwhelming selection, which makes choosing the right apps increasingly challenging for users. Overall, information about the quality of apps used in a clinical setting is limited^7^ and there is a lack of consensus on the methodology for app quality evaluation.^8,9^ Overcoming these limitations could lead to increased confidence in the credibility and usefulness of mHealth apps among professionals and end-users.

The Mobile Application Rating Scale (MARS),^10^ developed in 2015, is a short, objective, and reliable tool for classifying and assessing the quality of health apps. It has been cross-culturally adapted and validated in several languages^11-13^ and successfully used to assess the quality of mHealth apps.^14-17^ However, it requires a certain degree of health and e-Health expertise to ensure reliable and objective quality rating scores. A later simplified version was developed and validated, resulting in the User Version of the Mobile Application Rating Scale (uMARS), which can be used in large-scale trials, or research with end-users.^18^ The usefulness of the uMARS in the evaluation of mobile apps incorporated in the clinical setting has been recently demonstrated.^19,20^

While there is still a notable lack of content diversity of the Turkish mHealth apps—most of them address making doctor appointments, fertility issues, and general wellness—the market is gradually growing. However, the slow adoption among users and health professionals limits their impact and effectiveness.^21^ Thus, there is an increasing need for a reliable, culturally valid instrument to facilitate quality evaluation and support the dissemination of Turkish health apps. The scale would allow professionals, researchers, and developers to conceptualize, evaluate, recommend, and disseminate health apps with increased confidence.

Urology is not an exception in this growing app market. In the frame of an international project developing and testing the utility of an App (“MyBPH”) to monitor urinary symptoms, treatment compliance, and outcomes of patients affected by lower bladder outlet obstruction (BOO), the International Consortium of the study aimed also to assess the quality and ultimately the patients' perception of the app as the later will determine its utility and diffusion. The tool chosen to qualitatively assess the “MyBPH” App was the uMARS, of which there was not yet any existing cross-cultural translation and validation of the original English version to the Turkish language. Consequently, the present study aimed to translate, adapt, and cross-culturally validate a Turkish language version of the uMARS in the frame of the above-mentioned MyBPH project.

## Material and Methods

The study consisted of a cross-sectional linguistic and cross-cultural validation of uMARS to the Turkish language.

### The uMARS

The uMARS consists of 20 items, organized in five subscales, to evaluate the quality of health apps. Every item is rated on a 5-point Likert scale ranging from 1 (poor) to 5 (excellent). Four subscales are objective and related to quality rating: engagement (5 items), functionality (4 items), aesthetics (3 items), information (4 items), and one subscale evaluates app quality subjectively (4 items). A further subscale of 6 items measures the user-perceived impact of the evaluated app.^18^

An ethics committee approval was not necessary as this article was the result of the Turkish translation and validation of an already in-use international questionnaire. Voluntary participants had given their oral consent while answering the questions in the questionnaire so separate written consent was not required. Also, the questionnaire did not contain any medical information.

### Cross-Cultural Adaptation and Translation

**Figure 1.**
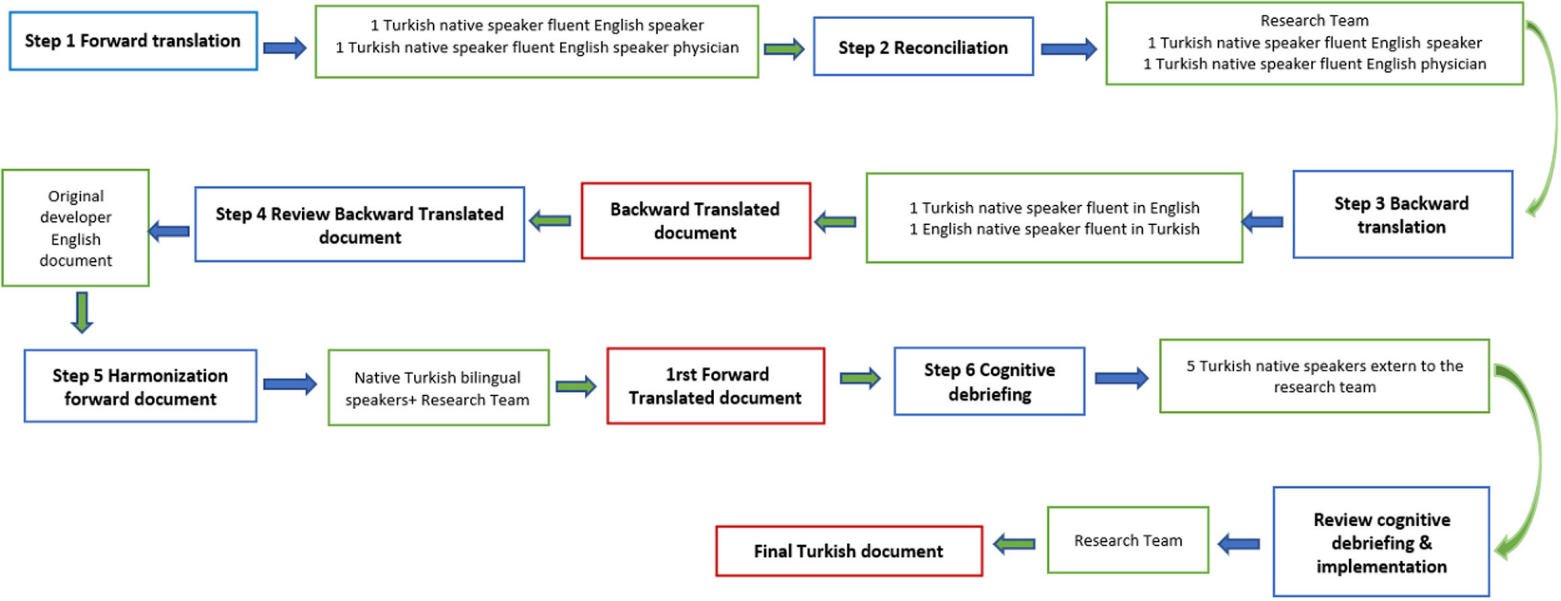
Schematic description of the validation process.

The methodology to complete the Turkish translation and cross-cultural validation of the English uMARS followed the World Health Organization guidelines,^22^ consisting of forwarding and backward translation, pilot testing, and accuracy evaluation to produce an accurate translation of the original scale (Figure 1).

A multidisciplinary team including researchers, health experts, and a language professional (with a degree in English literature) constituted the team involved in the project. The translation and adaptation involved the following process: (i) the English version of uMARS was independently translated to the Turkish language by two native Turkish bilingual speakers fluent in English (one physician and one non-medical professional); (ii) these two Turkish translations were discussed among the research team including the 2 original Turkish native bilingual speakers for harmonization purposes resulting in a single Turkish translation keeping the meaning the closest possible to the original English version; (iii) this Turkish version was then back-translated into English by two different bilingual researchers; (iv) the result was compared to the original English version of the original English scale and their feedback was implemented; (v) the final Turkish version was then reviewed by 5 Turkish-speaking individuals external to the research group. They provided feedback on their understanding of the items and the appropriateness of the Turkish language. Their comments and suggestions were discussed among the research group; and (vi) the necessary modifications to approximate the Turkish version to keep the English meaning were done based on the comments of the external revision resulting in the final Turkish version of the uMARS ready for testing (Addendum 1).

### Selection of the Mobile App for Validation

- Once the Turkish language translation was ready, a mobile app was selected according to the following inclusion criteria:

- Available in Google Play and Apple Store.
- Free of charge.
- Targeted at adults (≥18 years old) of any gender.
- Containing at least some features that allow fulfilling the core of the interaction to the medical counseling and intervention process (e.g., question and response, interchange of pictures or reports, and numerical results).
- Available in the Turkish language.

At study inception, there were 529 apps in the Turkish language in the category Health & Fitness. Apps were categorized in:

- *Specific gender or medical condition* (40.1%) focusing on specific medical conditions requiring expertise, baby care, or gender-biased in nature (e.g., ovulation control)
- Fitness or well-being (57.8%) (e.g., yoga or weight loss)
- Hospital or doctors’ appointments exclusively (1.9%)

Most of these apps required either knowledge of the condition or targeted a specific motivated population and their access was not for free. After discussion and consensus, the research team chose “WhatsApp”—a general communication app, accessible free of charge, and widely used across Turkey—for the validation testing. This choice was based on the presumption that WhatsApp can mimic the doctor–patient, therapist–client interaction apps, and that it was or could easily be used at least 1 month before the initiation of the first evaluation. Lastly, the team internally trialed the uMARS and thought that the uMARS scale could be applied to other types of apps, as long as they include at least some mHealth characteristics.

### Testing Population Sample

The population for validation was selected among the possible “end-users” of the uMARS. The sample size necessary was estimated at 50-100 individuals after discussion with the first author of the original uMARS.^18^

A call was made among fifth-year medical students of the Medipol University and medical workers of the Medipol University Hospital excluding physicians and the general population from the researchers’ networks. All participants were native Turkish speakers with at least secondary school graduations. The Turkish translation of the uMARS was distributed by email to all participants or paper-printed on demand.

Each participant was asked to use (or have used) the app for at least 1 month before completing the Turkish uMARS twice (4-6 weeks apart). They were instructed on completing the questionnaire individually and without help. Participants emailed or handed in their scores at the beginning and the end of the evaluation period. For each round, a reminder was sent 2 weeks after the initial distribution of the Turkish uMARS.

### Data Analysis

The internal consistency of the uMARS subscales and total score were calculated using Cronbach's *α*. Test-retest reliabilities were calculated for the subscales and total scores of the uMARS after the second round (i.e., a test-retest period of approximately 1 month). The within-group agreement (*r*_WG_ index) was chosen due to the skewness of the responses.^21,22^ Although the *r*_WG_ index is more a measure of agreement than a reliability index, it was preferred over the more commonly used the intraclass correlation (ICC) index because of the low variance in the recorded answers range.^23^ The vast majority of participants used a smaller range (3, 4, 5), therefore a unit in change is rather large within the overall range of answers (1-5). Furthermore, *r*_WG _is expected to perform better in Likert scale measurements and especially when a single group of participants is used than ICC.^24^

Levels of interrater agreement are: lack of agreement = 0.00 to 0.30; weak agreement 0.31 to 0.50; moderate agreement 0.51 to 0.70; strong agreement 0.71 to 0.90 and very strong agreement 0.91 to 1.00.^24^

Statistical analyses of the data were done by using the SPSS version 23 (IBM SPSS Corp.; Armonk, NY, USA).

## Results

The Turkish uMARS was distributed by email on December 23, 2019 to a sample group of 111 participants, all ≥18 years old. The last questionnaire of the second round was received on May 4, 2020.

Overall, 83 (74.7%) participants completed the first round after at least 1 month of app use and 74 (66.6% %) completed the second round (i.e., a test-retest period of approximately 1 month). The characteristics of the responders are described in Table 1. All the responders were fluent in spoken and written Turkish language.

Mean scores were highly consistent over time: for section A (*engagement*) they were 3.90 and 3.94 for the first round and second round, respectively; for section B (*functionality*) they were 4.28 and 4.22, respectively; for section C (*aesthetics*) they were 3.98 and 4.08, respectively; and for section D (*information*) 3.80 and 3.83, respectively. The mean *total mean score* (A+B+C+D/4), 3.99 for the first and second rounds (Table 2).

### uMARS Internal Consistency and Test-Retest Reliability

Consistency was calculated based on the first-round score (83 participants). The total uMARS score had a good internal consistency (Cronbach’s *α* = 0.87). Internal consistencies of its subscales were also acceptable (engagement *α* = 0.71; functionality *α* = 0.78; aesthetics *α* = 0.71; information *α* = 0.73) with a notable exception regarding satisfaction *α* that equaled 0.46, which is to be expected from a subjective scale.

Within-group agreement (*r*_WG_ index) was strong for all subscales and the total uMARS score (Table 2). The value of the item concerning payment was the only item negatively correlated.

## Discussion

We hereby present the development and validation of the cross-cultural adaptation of the Turkish uMARS. The results of this study indicate that the scale has a good internal consistency and test-retest reliability. Our data show that the 4 objective subscales of the Turkish version of the uMARS reflect the degree of correlation and strong agreement between measures or reliability. Most of the scores for all objective subscales also present strong reliability and validity levels. The satisfaction subscale was strongly influenced by large disparities in participant answers to question (#19) regarding the willingness to pay for the app. Interrater reliability within the group was equally high for all the questions except for question #19. Indeed, the question “*would you pay for this app*?” was the only one that affected the *r*_WG_ of the subjective quality subscale and had a negative interrater agreement. As the app chosen for validation is broadly used and freely available, one would expect a high level of concordance between the two response rounds and among respondents. However, willingness to pay highly depends on socioeconomic variables, the specific context of the consumer, and the service provided.^24^ However, our study participants ranged in socioeconomic status and, their ability or willingness to pay for an app may have been variable. Furthermore, considering the time frame of the first and second response rounds (beginning of 2020 with the ensuing COVID-19 pandemic), singular social, health, and economic factors may have played an additional and distinctive role in the intra- and inter-variability responses.

Previous research suggests that the dissemination of health apps without a proper quality evaluation may result in negative consequences for users (patients) and professionals (medical personnel).^25,26^ Although there are several instruments designed to evaluate the quality of medical apps, a clear definition of the theoretical framework to test quality is yet lacking and a wide heterogeneity exists in the criteria used to determine app quality.^27^ In this setting, uMARS offers a clear definition of the theoretical framework to test medical apps’ quality and has been translated to several languages and externally validated.^28-30^ In line with previous linguistic validations, the Turkish translation of the uMARS aims to bridge the gap between developers and end-users. It can be used for consumer research, as well as a checklist by mHealth developers in ensuring the high quality of their products.

In the frame of a study on the usefulness and effectiveness of a benign prostatic hyperplasia (MyBPH) app, trialed simultaneously in several countries, we needed an instrument to assess the quality of our app in Turkish.^21^ The choice of the uMARS as a quality measuring instrument was based on its robustness and simplicity when compared to other scales specifically designed to assess the quality of a medical app.^18^ Moreover, it is designed for end-users irrespective of expertise.

Hitherto, after following the necessary steps for linguistic translation and cultural adaptation for the validation of the Turkish uMARS version, we chose a broadly used app (“*WhatsApp*”) with which the general population was well acquainted. While the use of a non-medical app may be considered as a limitation, the main objective of this study was to translate, adapt, and cross-culturally validate a Turkish language version of the uMARS and it was not focused specifically on the scale’s apps in mHealth research. Rather than a limitation, the use of WhatsApp for cross-cultural validation can also be seen as a broadening of the uMARS uses. The original uMARS questions/items are general and refer to quality features that are not specific to a medical App and that apply to any App design in any field. Lastly, the chosen App was at that moment widely used by the Turkish population may decrease the burden of familiarizing with a new App, did not require specific knowledge from the evaluators, and was cost-free.

Limitations of our study include the possible negative influence on the response to subjective item #19 and the potential effect of the socioeconomic imbalance, which occurred during the COVID-19 pandemic on user scores of this item. However, as stated above, the uMARS permits for the subjectivity of this subscale and aims to correct for this through the computation of an overall subjective score, not reported as a component of the mean quality score of an app.

We believe that Turkish uMARS hereby presented is a reliable translation of the original scale and can be confidently used by researchers, developers, and health professionals and that it will facilitate further research and development of mHealth in Turkey offering professionals, researchers, and end-users a valid tool for and objective assessment of app’s quality in the Turkish language. Currently, further research is being conducted by our team, testing the properties of the scale when applied to an app for monitoring symptoms and treatment of Turkish patients with BOO caused by symptomatic MyBPH.

To the best of our knowledge, this study presents the first-ever translation of a user-targeted app quality measuring scale in the Turkish language. As research in this area continues to grow, further studies should explore the concurrent validity of the uMARS, compared to other app quality instruments focusing specifically on health apps.

The Turkish translation and cross-cultural validation of the uMARS showed good consistency (Cronbach’s *α* 0.87) and test-retest reliability (0.87). Overall, the test-retest agreement was strong for all the objective subscales of the uMARS (engagement, functionality, aesthetics, and information) with *r*_WG_ between 0.71 and 0.84. Although the subjective item (satisfaction) had a low consistency exclusively at the expenses of the question regarding payment, it also showed a strong test-retest agreement (*r*_WG_ 0.78).

## Figures and Tables

**Table 1.** Characteristics of the Population

Gender (Male/Female)	29 (34.9%)/54 (65.1%)
Median age (IQR, SD)	25 (23-35, 10.72)
Level of education University/post-graduate High or secondary studies	78 (94%) 5 (6%)
Occupation Medical students Healthcare workers* Other*	24 (28.9%) 43 (51.8%) 16 (19.3%)

*Excluding physicians.

**Table 2.** Test-Retest Agreement (*r*_WG_) Within-Group Agreement and Mean Scores of Subscales of the in the First and Second Rounds of Evaluation

Subscale/Item		*r*_WG_	Mean (SD) First Round	Mean (SD) Second Round
Engagement		0.81	3.90 (0.66)	3.94 (0.64)
1	Entertainment	0.65	3.94 (0.92)	4.00 (0.89)
2	Interest	0.46	3.59 (1.14)	3.82 (0.97)
3	Customization	0.61	3.90 (1.00)	3.91 (0.91)
4	Interactivity	0.45	3.89 (1.02)	3.91 (0.98)
5	Target group	0.58	4.24 (0.67)	4.09 (0.76)
Functionality		0.84	4.28 (0.54)	4.21 (0.37)
6	Performance	0.60	4.17 (0.87)	4.11 (0.80)
7	Ease of use	0.69	4.46 (0.74)	4.38 (0.66)
8	Navigation	0.73	4.22 (0.61)	4.22 (0.60)
9	Gestural design	0.72	4.19 (0.71)	4.03 (0.60)
Aesthetics		0.80	3.98 (0.56)	4.08 (0.49)
10	Layout	0.71	4.13 (0.71)	4.07 (0.73)
11	Graphics	0.66	3.92 (0.68)	4.00 (0.72)
12	Visual appeal	0.60	3.80 (0.76)	4.12 (0.74)
Information		0.71	3.80 (0.73)	3.83 (0.65)
13	Quality of information	0.46	3.96 (0.91)	3.65 (1.01)
14	Quantity of information	0.44	3.74 (1.04)	3.74 (1.01)
15	Visual information	0.60	4.11 (0.81)	4.09 (0.85)
16	Credibility of source	0.30	3.47 (1.01)	3.64 (1.18)
Total UMARS		0.87	3.99 (0.50)	3.99 (0.48)
Subjective items		0.78	4.01 (0.70)	4.01 (066)
17	Would you recommend this app	0.69	4.39 (076)	4.41 (0.70)
18	How many times do you think you will use the app?	0.72	4.73 (0.66)	4.55 (0.81)
19	Would you pay for this app?	-0.23	2.66 (1.77)	2.81 (1.56)
20	What is your overall rating of this app?	0.59	4.19 (0.94)	4.28 (0.73)

## Kullanım Talimatları

### Kullanıcılar;

1. Uygulamayı etraflıca en az 10 dakika süreyle kullanmalı,
2. Kullanımının ne kadar kolay olduğuna, ne kadar iyi işlev gördüğüne ve amacına ne kadar uygun olduğuna karar vermeli,
3. Uygulama ayarlarını, geliştirici bilgilerini, dış bağlantılarını, güvenlik özelliklerini vs. değerlendirmelidir.

**Puanlama**

A: Ortalama katılım puanı=

B: Ortalama işlevsellik puanı=

C: Ortalama estetik puanı=

D: Ortalama puanı*=

* Boş bırakılan veya “uygun değil” soruları ortalama hesabı dışında tutun

**Uygulama Kalitesi ortalama puanı-----------------=**A+B+C+D /4

Uygulama öznel kalitesi, araştırmanın amaçlarına bağlı olarak, bireysel öğeler veya ortalama bir puan olarak raporlanabilir.

Algılanan etki öğeleri, hedef sağlık davranışlarına ilişkin uygulamanın kullanıcı bilgisi, davranışları ve niyetleri üzerindeki algılanan etkisine yönelik düzenlenebilir ve bilgi toplama amaçlı kullanılabilir.

**Mobil Uygulama Değerlendirme Ölçeği:**

**Kullanıcı versiyonu (uMARS)**

**Uygulama Adı: ------------------**

Derecelendirdiğiniz uygulamanın kalitesini en doğru şekilde temsil eden sayıyı daire içine alın. Tüm öğeler 1'den 5’e giden bir ölçekte derecelendirilmiştir. “1. Yetersiz”- “5. Mükemmel" anlamını taşır. Uygulama bileşeni alakasızsa, “uygun değil” seçeneğini işaretleyin.

## Uygulama Kalitesi Puanlaması

### BÖLÜM A

**Katılım- eğlenceli, ilginç, özelleştirilebilir, etkileşimli ve istemleri olması (Uyarılar, mesajlar, hatırlatıcılar, geribildirimler göndermesi; paylaşıma uygunluk)**

1. **Eğlence: Uygulama kullanımı eğlenceli mi? Benzer uygulamalardan daha eğlenceli bileşenleri var mı?**
  1. Donuk, hiçbir şekilde eğlenceli değil
  2. Çok sıkıcı
  3. İdare eder, kısa süreliğine (<5 dk.) kullanıcıyı eğlendirecek nitelikte
  4. Oldukça eğlenceli, bir süreliğine (5-10dk) kullanıcıyı eğlendirir
  5. Çok eğlenceli, sürekli tekrar kullanımı teşvik eder.
2. **İlginçlik: Uygulama kullanımı ilginç mi? Benzer uygulamalara göre bilgiyi daha ilgi çekici şekilde mi sunuyor?**
  1. Hiçbir şekilde ilginç değil
  2. Genel olarak ilginç değil
  3. İdare eder ne ilginç ne değil, kullanıcıyı kısa süreliğine idare eder (<5 dk.)
  4. Oldukça ilginç, kullanıcıyı bir süreliğine (5-10dk) idare eder.
  5. Çok ilginç, kullanıcıyı tekrar kullanıma teşvik eder.
  1. **Kişiselleştirme: İsteğinize göre ayarları (ör. Ses, içerik ve bildirimler) ve tercihleri değiştirmenize izin veriyor mu?**

1. Hiçbir şekilde kişiselleştirmeye izin yok veya her seferinde tekrardan ayar gerekiyor 2. Çok kısıtlı ayara izin veriyor ve uygulama işlevlerini sınırlıyor 3. Düzgün çalışması için temel ayarlara izin var 4. Kişiselleştirme için belli sayıda seçenek var 5. Kullanıcının özelliklerine / tercihlerine göre tam olarak uyarlama imkanı verir, tüm ayarları hatırlar

1. **Etkileşim: Kullanıcı girişine, geribildirime, uyarılara (hatırlatıcılar, paylaşım seçenekleri, bildirimler) izin veriyor mu?**

1. Hiç interaktif özellik yok ve/veya kullanıcı girişine cevap vermiyor

2. Bazı etkileşim öğeleri var, fakat sınırlı ve uygulamanın işlevselliğini sınırlıyor

3. Düzgün çalışması için temel interaktif özellikler mevcut

4. Bir dizi interaktif özellik, geribildirim ve kullanıcı girişi içerir

5. İnteraktif özellikleri sayesinde üst seviye geribildirim ve kullanıcı girişi sağlar.

**5. Hedef Kitle: Uygulama içeriği (görseller, dil, tasarım) hedef kitleye uygun mu?**

1. Tamamen uygunsuz, belirsiz veya kafa karıştırıcı

2. Oldukça uygunsuz, belirsiz veya kafa karıştırıcı

3. Yeterli ancak özellikle hedef kitleye özgü değil. Bazen yetersiz/belirsiz/karışık olabilir

4. Bazı eksiklikler haricinde hedef kitleye özgü

5. Tamamen hedef kitleye özgü, eksik yok.

### BÖLÜM B

**İşlevsellik- Uygulamanın işlevselliği, öğrenme kolaylığı, uygulama içinde gezinme, akış mantığı ve uygulamanın el hareketleri ile kullanımı**

**6. Performans: Uygulama özellikleri(işlevleri) ve bileşenleri(tuşlar/menüler) ne kadar doğru/hızlı?**

1. Uygulama bozuk; yetersiz ya da hiç tepki yok (ör. Çökme/hata/bozuk özellik vs.)

2. Bazı işlevler çalışıyor ancak yavaşlama var veya önemli teknik sorunlar var

3. Uygulama genel olarak çalışıyor. Bazı teknik sorunlar giderilmeli veya bazen yavaşlama var

4. Genel olarak çalışan programda ufak aksamalar var

5. Mükemmel/zamanında tepkiler; hiçbir teknik arıza yok veya “Kalan yükleme süresi” uyarısı gösteriyor (varsa).

1. **Kullanım kolaylığı: Uygulamayı kullanmayı öğrenmek ne kadar kolay; menü etiketleri, simgeler ve talimatlar ne kadar net?**
2. Talimatlar sınırlı veya hiç yok; menü etiketleri, simgeler karışık
3. Alışmak çok uzun zaman alıyor
4. Alışmak biraz zaman alıyor
5. Öğrenmesi kolay (veya düzgün açıklamalar var)
6. Uygulama hemen kullanılabiliyor, sezgisel ve basit (açıklama gerektirmiyor).

**8. Gezinme: Ekranlar arası geçiş anlamlı mı? Uygulama ekranlar arası gerekli bağlantılara sahip mi?**

1. Ekranlar arası hiçbir şekilde mantıklı bağlantı yok/gezinme çok güç

2. Uzun süre uğraştıktan sonra anlaşılabiliyor

3. Biraz uğraştıktan sonra anlaşılabiliyor 4. Anlaması / gezinmesi kolay 5.Tamamen mükemmel, kolay, açık ve sezgisel ekran akışı var ve/veya kısa yollar mevcut.

**9. Hareketli tasarım: Dokunma/kaydırma/tıklama/zumlama anlamlı mı? Tüm bunlar ekranlarda/bileşenlerde tutarlı mı?**

1. Tamamen tutarsız/karışık

2. Genellikle tutarsız/karışık

3. İdare eder, bazı tutarsızlıklar/kafa karıştırıcı unsurlar var

4. Gözardı edilebilecek sorunlar hariç genel olarak tutarlı/sezgisel

5. Tamamen tutarlı ve sezgisel

### BÖLÜM C

**Estetik- Grafik tasarımı, Genel görsel çekicilik, renk şeması ve stil tutarlılığı**

**10. Düzen: Ekrandaki tuşların, simgelerin, menülerin ve içeriğin dizilimi ve boyutları uygun mu?**

1. Çok kötü tasarım, darmadağın; bazı seçenekleri seçmek, bulmak, görmek, okumak imkânsız

2. Kötü tasarım, rastgele, belirsiz; bazı seçenekleri seçmek, bulmak, görmek, okumak zor

3. Yeterli, maddeleri seçmek, bulmak, görmek, okumak ile ilgili bazı sorunlar var

4. Çoğunlukla açık; maddeleri seçmek, bulmak, görmek, okumak kolay

5. Profesyonel, basit, açık, düzgün, mantıklı sıralı

**11. Grafikler: Tuşlar, simgeler, menüler ve içerik için kullanılan grafikler ne kadar kaliteli?**

1. Amatör grafikler, çok kötü görsel tasarım-orantısız, şeklen uyumsuz

2. Düşük kalite/çözünürlüklü grafikler; düşük kalite görsel tasarım-orantısız

3. Orta kalite grafikler ve görsel tasarım (şeklen genel olarak tutarlı)

4. Yüksek kalite/çözünürlüklü grafikler ve görsel tasarım- şeklen çoğunlukla uyumlu ve orantılı

5. Çok yüksek kalite/çözünürlüklü grafikler ve görsel tasarım-şeklen tamamen uyumlu ve orantılı

**12. Görsel çekicilik: Uygulama nasıl görünüyor?**

1. Çirkin, hoş olmayan, kötü tasarım, çatışan ve uyumsuz renkler

2. Kötü- kötü tasarım, kötü renkler, sıkıcı görsellik

3. İdare eder, ortalama ne iyi ne kötü

4. Hoş-düzgün grafikler-tutarlı ve profesyonelce tasarlanmış

5. Güzel- çok çekici, unutulmaz, öne çıkan; uygulama özelliklerini öne çıkaran renkler var.

### BÖLÜM D

**Bilgi-Düzgün bir kaynaktan yüksek kalitede bilgiler (yazı, geribildirim, kararlar, kaynaklar) içerir**

**13. Bilgi kalitesi: Uygulamanın içeriği doğru düzgün yazılmış mı ve uygulamanın ana fikriyle ilgili mi?**

Uygun değil: Uygulamanın içerisinde herhangi bir açıklama yok

1. İlgisiz/uygunsuz/tutarsız/yanlış bilgiler

2. Zayıf, neredeyse tamamen ilgisiz/uygunsuz/tutarsız/olası yanlış bilgiler

3. Kısmen ilgili/uygun/tutarlı/olası doğru bilgiler

4. İlgili/uygun/tutarlı/doğru bilgiler 5.Çok ilgili/çok uygun/çok tutarlı/doğru bilgiler

**14. Bilgi miktarı: Uygulamadaki bilgiler kapsamlı mı özlü mü?**

Uygun değil: Uygulamada herhangi bir bilgi yok

1. Çok sınırlı veya aşırı fazla

2. Yetersiz veya bir miktar fazla

3. Yeterli ancak ne kapsamlı ne de özlü bilgiler var

4. Geniş bir yelpazede bilgi sunuyor ancak bazı boşluklar veya gereksiz detaylar var veya daha fazla bilgiye ve kaynağa bağlantı yok

5. Kapsamlı ve özlü bilgiler mevcut, daha fazla bilgiye ve kaynağa bağlantı var.

**15. Görsel bilgiler: Kavramların görsel açıklaması- çizelgeler / grafikler / görüntüler / videolar vb.- açık, mantıklı, doğru mu?**

Uygun değil: Uygulamada herhangi bir görsel bilgi yok (örn. Sadece ses veya yazı içeriyor)

1. Tamamen belirsiz/karışık/yanlış veya gerekli bilgiler kayıp

2. Çoğunlukla belirsiz/karışık/yanlış

3. Yeterli ancak bazen belirsiz/karışık/yanlış

4. Çoğunlukla açık/mantıklı/doğru ancak bazen göz ardı edilebilen eksiklikler var

5. Tamamen açık/mantıklı/doğru

**16. Kaynağın güvenilirliği: Uygulama içindeki bilgiler güvenilir bir kaynaktan geliyor mu?**

Uygun değil: Uygulama içinde bilgi yok.

1. Şüpheli kaynak

2. Zayıf güvenilirlik

3. Şüpheli değil ancak kaynağın meşruluğu tartışılır

4. Büyük olasılıkla güvenilir bir kaynağa bağlı

5. Tamamen güvenilir/uzman bir kaynağa bağlı

**Uygulama öznel kalitesi**

### BÖLÜM E

**17. Bu uygulamayı faydalanabilecek kişilere önerir misiniz?**

1. Hiçbir zaman, bu uygulamayı kimseye önermem

2. Bu uygulamayı çok az insana önerebilirim

3. Belki, bu uygulamayı bazı insanlara önerebilirim

4. Bu uygulamayı çoğu kişiye önerebilirim.

5. Kesinlikle, bu uygulamayı herkese öneririm.

**18. Bu uygulamayı önümüzdeki 12 ay içinde kaç kez kullanacağınızı düşünüyorsunuz? (Eğer sizle alakalı ise)**

1. Hiç

2. 1-2 kez

3. 3-10 kez

4. 10-50 kez

5. 50’den fazla kez

**19. Bu uygulama için ücret öder miydiniz?**

1. Asla ödemem

2.

3.

4.

5. Kesinlikle öderim

**20. Bu uygulama için değerlendirmeniz(yıldızınız) nasıl?**

1. * Kullandığım en kötü uygulamalardan biri

2.**

3.*** Orta halli

4.****

5.*****Kullandığım en iyi uygulamalardan biri

**Algılanan Etki**

### BÖLÜM F

**1. Farkındalık-Bu uygulama benim bu sağlık davranışıyla ilgili dikkatimi arttırdı**

Tamamen katılmıyorum............................................................................................Tamamen katılıyorum

.................1.......................................2.......................................3.......................................4.......................................5

**2. Bilinç- Bu uygulama benim bu sağlık davranışıyla ilgili bilincimi arttırdı**

Tamamen katılmıyorum............................................................................................Tamamen katılıyorum

.................1.......................................2.......................................3.......................................4.......................................5

**3. Tutum- Bu uygulama benim bu sağlık davranışıyla ilgili tutumumu değiştirdi**

Tamamen katılmıyorum............................................................................................Tamamen katılıyorum

.................1.......................................2.......................................3.......................................4.......................................5

**4. Değişim isteği- Bu uygulama benim bu sağlık davranışıyla ilgili değişim isteğimi arttırdı**

Tamamen katılmıyorum............................................................................................Tamamen katılıyorum

.................1.......................................2.......................................3.......................................4.......................................5

**5. Yardım arayışı- Bu uygulama beni bu sağlık davranışı ile ilgili olarak yardım aramam için cesaretlendirdi**

Tamamen katılmıyorum............................................................................................Tamamen katılıyorum

.................1.......................................2.......................................3.......................................4.......................................5

**6. Davranış değişimi- Bu uygulamanın kullanımı sağlık davranışımı artıracak / azaltacaktır**

Tamamen katılmıyorum............................................................................................Tamamen katılıyorum

.................1.......................................2.......................................3.......................................4.......................................5

**Uygulama ile ilgili görüş ve önerileriniz**

**TEŞEKKÜRLER!**
